# Supplementary material for: Causal Relationship between Aging and Anorexia Nervosa: A White-Matter-Microstructure-Mediated Mendelian Randomization Analysis
Source: Biomedicines. 2024 Aug 16;12(8):1874. doi: 10.3390/biomedicines12081874 (PMC11351342; doi:10.3390/biomedicines12081874)
Supplement: Supplementary file 1 [file biomedicines-12-01874-s001.zip › Supplementary Figure/Figure S8/Leaveoneout plot of MR analysis for TL to ubm-b-1630.pdf]

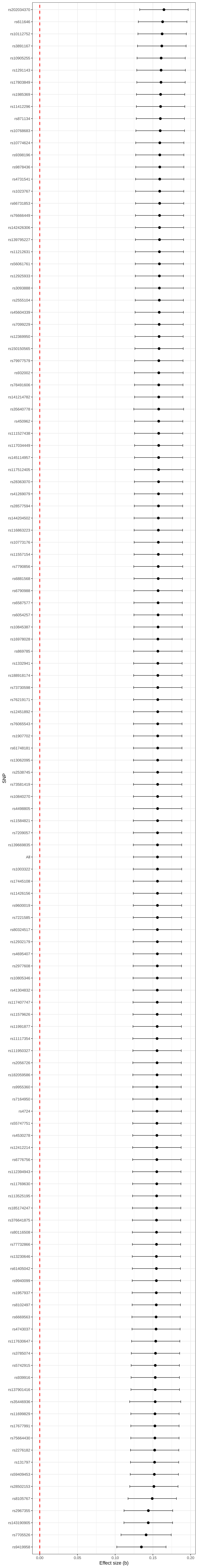
rs202034370
rs611646
rs10112752
rs3891167
rs10905255
rs1291143
rs17803849
rs1985369
rs11412296
rs871134
rs10768683
rs10774624
rs9398196
rs9878436
rs4731541
rs1023767
rs66731853
rs76666449
rs142426306
rs139795227
rs11212631
rs56061761
rs12925933
rs3093888
rs2555104
rs45604339
rs7099229
rs12369950
rs150150565
rs79977579
rs932002
rs78491606
rs141214782
rs35640778
rs450962
rs111527438
rs117034449
rs145114957
rs117512405
rs28363070
rs41269079
rs28577594
rs144204502
rs116863223
rs10773176
rs11557154
rs7790856
rs6881568
rs6790988
rs6587577
rs6054257
rs10845387
rs16978028
rs869785
rs1332941
rs188918174
rs73730598
rs76219171
rs12451892
rs76065543
rs1907702
rs61748181
rs13062095
rs2538745
rs73581419
rs10840270
rs4498805
rs11584821
rs7209057
rs139669835
All
rs1003322
rs17445108
rs11426156
rs9600019
rs7221585
rs80324517
rs12932179
rs4695407
rs2977608
rs10805346
rs41304832
rs117407747
rs11579626
rs11991877
rs11117354
rs111950327
rs2056726
rs182059586
rs9955360
rs7164950
rs4724
rs55747751
rs4530278
rs12412214
rs6776756
rs112394943
rs11769630
rs113525195
rs185174247
rs376641875
rs80116508
rs77732866
rs13230646
rs61405042
rs9940099
rs1957937
rs8102497
rs6669563
rs4743037
rs117630647
rs3785074
rs5742915
rs939916
rs137901416
rs35446936
rs11699829
rs17677991
rs75664430
rs2276182
rs131797
rs59409453
rs28502153
rs8105767
rs2967355
rs143190905
rs7705526
rs9419958
SNP
0.00
0.05
0.10
0.15
0.20
Effect size (b)
